# 

**Published:** 1844-10

**Authors:** 


### WESTERN JOURNAL

OF

### MEDICINE AND SURGERY.

### EDITORS.

---

DANIEL DRAKE, M. D.,

PROFESSOR OF PATHOLOGY AND THE PRACTICE OF MEDICINE IN THE MEDICAL INSTITUTE OF LOUISVILLE:

LUNSFORD P. YANDELL, M. D.,

PROFESSOR OF CHEMISTRY AND PHARMACY IN THE SAME:

THOMAS W. COLESCOTT, M. D.

### TO THE SUBSCRIBERS OF THIS JOURNAL.

The publishers address you in this number, on the subject of your delinquencies. They cannot go on with the Journal unless you pay. We appeal to you to come forward promptly and place the work on a sure footing. To many of you we feel that we can thus appeal as friends, and we have full confidence that the appeal will not be made in vain.

### TO SUBSCRIBERS.

We regret to find that, notwithstanding unusual expenditures upon the present volume of this Journal and its very material improvement, our subscribers have this year been more than usually remiss. We would give notice that all who may not remit promptly shall be forthwith and constantly harrassed with every form of dun until they are worried out. Their names shall appear in a black list on the back of the Journal, and, simultaneously, they shall be called upon and persecuted by duns of every kind, travelling agents, local agents, constables, sheriffs, and catchpoles. We are shamefully deprived of our dues, and we mean to have justice done us.

PRENTICE & WEISSINGER.

☞Receipt by mail, at our risk, under frank of post-master or post-paid.

*Receipts of Medical Journal for August and September.*

| | |
|---|---|
| Dr. Loving, Paris, Tenn. | $5 00 |
| J. H. Shackleford, Vernon, Ala. | 5 00 |
| T. S. Headen, Lynnville, Ill. | 10 00 |
| J. Harvey, Harveysburg, O. | 5 00 |
| S. Riggin, Hillsboro, Ky. | 5 00 |
| J. Truman, Wilmington. O. | 5 00 |
| S. Slaughter, Chineyville, La. | 12 16 |
| G. W. Forman, High Grove, Ky. | 5 00 |
| J. A. Smith, St. Martinsville, La. | 3 00 |
| Baker & Packard, Kivsawqua, Iowa. | 5 00 |
| Dr. Summers, County | 10 00 |
| P. Herrod, Pleasant Shade, Tenn. | 5 00 |
| Dr. A. B. Dodd, Bolivar, Miss. | 2 62 |

